# SOMEBODY ELSE TO THINK ABOUT BESIDES MYSELF: VOLUNTEER EXPERIENCES WITH THE CARING CALLERS PROGRAM DURING COVID-19

**DOI:** 10.1093/geroni/igac059.2348

**Published:** 2022-12-20

**Authors:** Noelle Fields, Kathy Lee, Jessica Cassidy

**Affiliations:** The University of Texas at Arlington, Arlington, Texas, United States; University of Texas at Arlington, Coppell, Texas, United States; University of Texas at Arlington, Arlington, Texas, United States

## Abstract

Social distancing during COVID-19 had significant implications for older adults, a subpopulation already at greater risk of social isolation and loneliness prior to the pandemic. Emerging evidence showed promise for implementing peer- and volunteer-led, telephone-based support programs in collaboration with community agencies to reach older adults at-risk for loneliness. Given a need for research in this area, the Caring Callers Program was developed as a telephone-based intervention using Senior Companion volunteers for at-risk older adults in a large metropolitan area in Texas. Volunteers were provided training via zoom to prepare them to provide supportive, weekly calls as well as to share community resources with their Caring Callers clients. The purpose of this study was to qualitatively explore the experiences of Senior Companion volunteers (N = 20) in the Caring Caller Program through the lens of productive aging. Rapid and Rigorous Qualitative Date Analysis (RADaR) was used to analyze the data and yielded four themes: 1) reciprocity, reflecting how volunteers described the program mutually benefited their feelings of well-being as well as, those contacted.; 2) purposeful use of time, illustrating how the volunteers perceived their role as meaningful and fulfilled their value for civic duty; 3) learning new skills, such as active listening skills; and 4) gaining perspective, capturing how the volunteers gained new inspiration from their experiences in the program. Recommendations for future research and strategies for designing peer-led, telephone-based interventions to promote social connectedness among both older volunteers and socially isolated older adults are offered.

